# Evaluation of vascular graft infection following Bentall surgery using 18F‐FDG PET/CT scan: A pediatric case report

**DOI:** 10.1002/ccr3.8396

**Published:** 2023-12-29

**Authors:** Mehrosadat Alavi, Maryam Abdinejad, Mehdi Rezaei, Alireza Moaref

**Affiliations:** ^1^ Ionizing and Non‐Ionizing Radiation Protection Research Center School of Paramedicine Sciences Shiraz University of Medical Sciences Shiraz Iran; ^2^ Department of Nuclear Medicine, School of Medicine Shiraz University of Medical Sciences Shiraz Iran; ^3^ Nuclear Medicine and Molecular Imaging Research Center Namazi Teaching Hospital, Shiraz University of Medical Sciences Shiraz Iran; ^4^ Department of Cardiology Fars‐Iranian Heart Association Fars Society of Internal Medicine Shiraz Iran; ^5^ Cardiovascular Research Center Shiraz University of Medical Sciences Shiraz Iran

**Keywords:** 18‐F‐FDG PET/CT scan, Bentall surgery, vascular graft infection

## Abstract

**Key Clinical Message:**

After a Bentall surgery, there is a small chance of developing a serious complication called vascular graft infection. 18F‐FDG PET/CT, a new and accurate diagnostic tool, can help detect it early, especially if the symptoms are unusual.

**Abstract:**

A 14‐year‐old boy who had undergone Bentall surgery 1 year prior presented with symptoms of fever, chills, loss of appetite, and weight loss over the course of a month. The initial Bentall surgery was performed due to an aneurysm of the thoracic aorta, along with severe aortic valve insufficiency and moderate aortic valve stenosis. The patient was referred to the PET/CT department for evaluation of possible endarteritis or infection of Dacron graft, which had been reported in trans‐esophageal echocardiography as suspicious findings. Despite normal blood tests, blood cultures, and other imaging modalities, the 18F‐FDG PET/CT confirmed the diagnosis of vascular graft infection. This diagnostic tool allowed for timely and appropriate treatment and prevention of possible complications.

## Graphical abstract

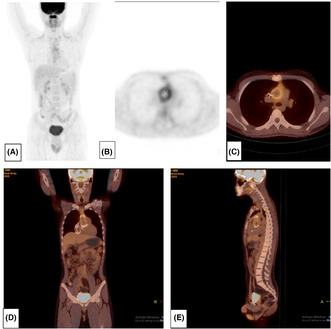

## 1 INTRODUCTION

The Bentall procedure is a documented technique used to repair congenital anomalies of the aortic valve, root, and ascending aorta.^1^ Vascular prosthetic graft infection is a rare but serious complication after surgery,^2^ with a prevalence of 0.5%–5%, and poses a significant challenge in diagnosis, particularly in cases of low‐grade infection.^3^

## 2 CASE PRESENTATION

A 14‐year‐old boy with a medical history of congenital bicuspid aortic valve presented with a syncopal attack along with hypotension, tachycardia, and dyspnea while playing football, and was diagnosed with thoracic aortic aneurysm, severe aortic valve insufficiency, and moderate aortic valve stenosis. A Bentall operation with a composite graft was performed, and a cardiac pacemaker was implanted due to the abnormal cardiac rhythm. During the recovery phase, pericardial effusion occurred, which was resolved with appropriate treatment. The patient was discharged in good condition.

One year after the surgery, he presented with fever, chills, loss of appetite, and weight loss. Blood tests, a blood culture, a chest x‐ray, and an abdominopelvic sonography were normal. However, a transthoracic echocardiography (TTE) showed equivocal findings. As a result, the patient was referred for a transesophageal echocardiography (TEE), which revealed that the ascending aorta had been replaced by a Dacron graft. The posterior aortic wall showed thickening with some echo‐free space that could be partially liquefied hematoma or periaortic inflammation.

**FIGURE 1:**
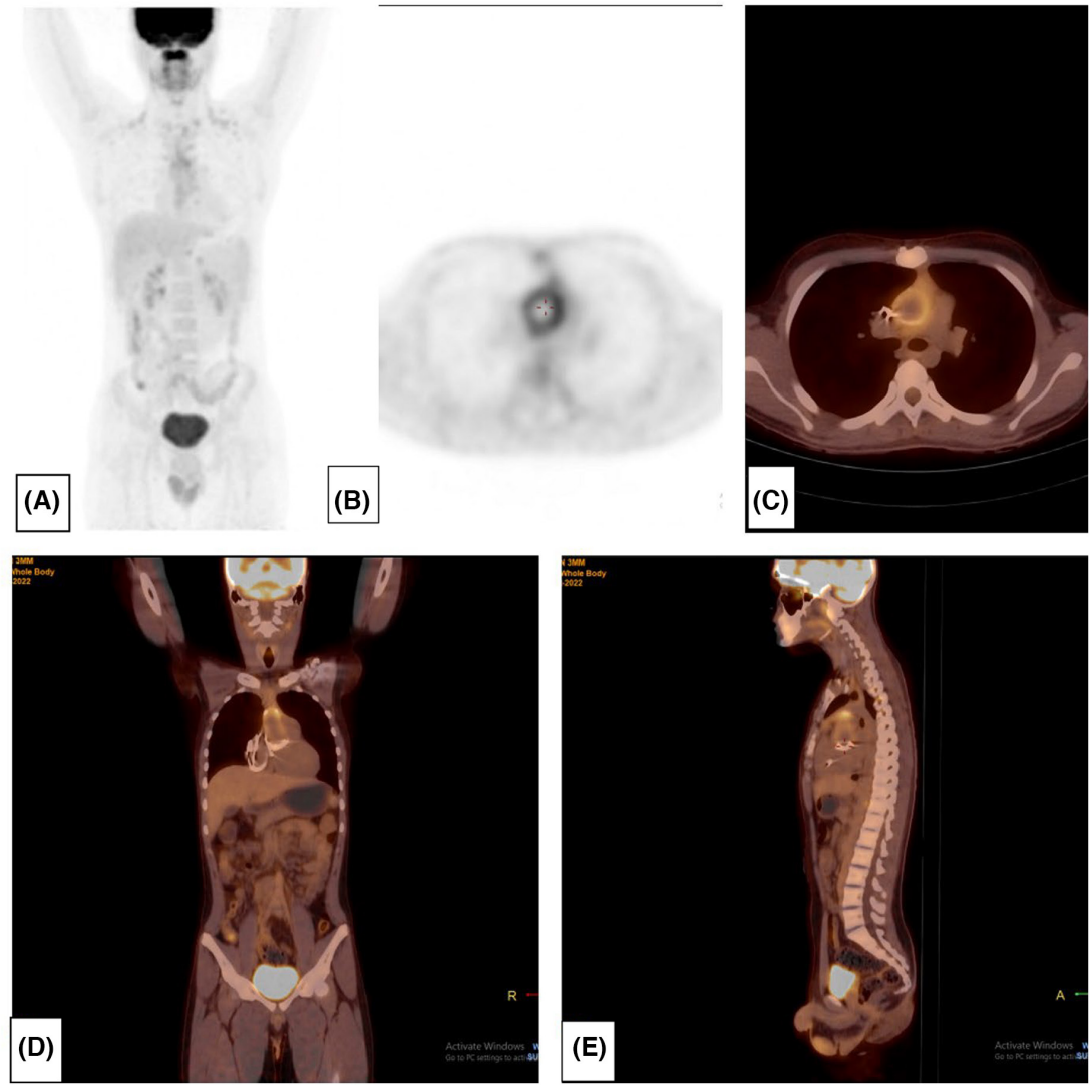
MIP (A), Trans‐axial PET (B), PET/CT (C), sagittal PET/CT (D) and Coronal PET/CT (E) images, showing increased 18F‐FDG uptake around the aortic root, that represented graft infection of the aorta.

Eventually, the patient was referred to PET/CT scan department. After a long‐term fast, as well as a low carbohydrate and high protein diet in the previous 24 h, a whole body 18F‐FDG PET/CT scan was performed 1 h after an IV injection of 285MBq 18F‐FDG, with a blood glucose level of 114 mg/dL at the time of injection. The scan revealed circumferential increased FDG uptake around the aortic root, extending up to the ascending aorta (SUV max:4.11), which represented an aortic graft infection (Figure 1).

Based on the PET/CT results, the patient was administered appropriate antibiotics such as vancomycin and gentamicin iv for 6 weeks. Currently, 2 weeks after discharge, the patient is in good health and without any specific complaints, and being monitored with oral antibiotics.

## 3 DISCUSSION

Signs and symptoms of prosthetic vascular infections are highly variable and non‐specific.^4^ CTA has been employed as a complementary diagnostic tool to diagnose diseases due to its high spatial resolution. However, its diagnostic sensitivity decreases in cases of low‐grade infection, with an overall sensitivity of only 55.5% compared to 94% in advanced graft infections.^5^ Conversely, studies on the use of MRI and 111‐In WBC scans to diagnose vascular graft infections have shown little significance.^6^

Infection and inflammation boost the metabolism of glucose in the cells, leading to increased uptake of FDG by inflamed cells. A study by Keidar et al. demonstrated that 18F‐FDG PET/CT has a sensitivity, specificity, positive predictive value, and negative predictive value of 93%, 91%, 88%, and 96%, respectively, for detecting vascular graft infection.^7^ However, the possibility of false‐positive results should always be considered.^5^ Nonetheless, using the 5‐point visual grading score in patients who have not received antibiotic treatment leads to a 100% diagnostic accuracy when diagnosing vascular graft infection using 18F‐FDG‐PET/CT.^8^

## 4 CONCLUSION

Vascular graft infection is a rare but serious complication that can occur following Bentall surgery. The 18F‐FDG PET/CT is a promising, new, and accurate diagnostic modality that is especially useful in cases with anomalous clinical presentations.

## AUTHOR CONTRIBUTIONS

**Mehrosadat Alavi:** Project administration; supervision; writing – review and editing. **Maryam Abdinejad:** Project administration; writing – review and editing. **Mehdi Rezaei:** Data curation; investigation; writing – original draft. **Alireza Moaref:** Data curation; investigation; writing – original draft.

## FUNDING INFORMATION

No funding was received for this study.

## CONFLICT OF INTEREST STATEMENT

The authors declare no conflicts of interest.

## CONSENT

Written informed consent was obtained from the patient to publish this report in accordance with the journal's patient consent policy.

## Data Availability

Data sharing not applicable to this article as no datasets were generated or analyzed during the current study.
